# Establishing and storing of deterministic quantum entanglement among three distant atomic ensembles

**DOI:** 10.1038/s41467-017-00809-9

**Published:** 2017-09-28

**Authors:** Zhihui Yan, Liang Wu, Xiaojun Jia, Yanhong Liu, Ruijie Deng, Shujing Li, Hai Wang, Changde Xie, Kunchi Peng

**Affiliations:** 1 State Key Laboratory of Quantum Optics and Quantum Optics Devices, Institute of Opto-Electronics, Shanxi University, Taiyuan, 030006 China; 2 Collaborative Innovation Center of Extreme Optics, Shanxi University, Taiyuan, 030006 China

## Abstract

It is crucial for the physical realization of quantum information networks to first establish entanglement among multiple space-separated quantum memories and then, at a user-controlled moment, to transfer the stored entanglement to quantum channels for distribution and conveyance of information. Here we present an experimental demonstration on generation, storage, and transfer of deterministic quantum entanglement among three spatially separated atomic ensembles. The off-line prepared multipartite entanglement of optical modes is mapped into three distant atomic ensembles to establish entanglement of atomic spin waves via electromagnetically induced transparency light–matter interaction. Then the stored atomic entanglement is transferred into a tripartite quadrature entangled state of light, which is space-separated and can be dynamically allocated to three quantum channels for conveying quantum information. The existence of entanglement among three released optical modes verifies that the system has the capacity to preserve multipartite entanglement. The presented protocol can be directly extended to larger quantum networks with more nodes.

## Abstract (web summary)

Continuous-variable encoding is a promising approach for quantum information and communication networks. Here, the authors show how to map entanglement from three spatial optical modes to three separated atomic samples via electromagnetically induced transparency, releasing it later on demand.

## Introduction

Flying photons or bright optical beams are the best natural quantum channels, while usually matter systems are employed for memories at quantum nodes^1, 2^. Single atoms^3, 4^, atomic ensembles^5–10^, trapped ions^11–13^, optomechanics^14–17^, superconductors^18^, solid-state systems^19–22^, and so on have been applied as quantum nodes. Especially, atomic ensembles are among the best candidates for quantum nodes to store and process quantum information due to the advantage of the collective enhancement of light–atom interaction^5–10^.

The entanglement of discrete quantum variables between two atomic ensembles has been experimentally achieved by means of Raman scattering approach^23, 24^ or transferring quantum states of entangled photons into two atomic systems^25–27^. In 2010, Kimble’s group demonstrated measurement-induced entanglement stored in four atomic memories and coherent transfer of the atomic entanglement to four photonic channels^28^. For the first time, their experiment proved that a multipartite entangled W state of atomic ensembles can be transferred into a photonic mode W state with the heralded entanglement and thus showed an advance in the distribution of multipartite entanglement across quantum networks. Besides above-mentioned schemes based on applying discrete quantum variables of single photons and atoms, continuous-variable (CV) regime provides another avenue toward the realization of quantum information tasks. To develop CV quantum information networks, CV entanglement between two macroscopic objects, i.e., atomic ensembles, has been investigated^29, 30^. CV entanglement of spin wave variances between two atomic ensembles has been experimentally realized via quantum non-demolition measurement^29^ and dissipation mechanism of atomic systems^30^, respectively. For implementing quantum computation^31^ and quantum communication^32^, entanglement has to be stored in atomic memories and then to be controllably released on demand. Quantum memories for squeezing and entanglement of light have been theoretically investigated^33, 34^, and the storage of CV entanglement between two atomic ensembles has been experimentally completed^35^. So far, all experiments on generation and storage of CV entanglement of atomic systems are concentrated between two ensembles^29, 30, 35^. In ref. ^35^, a displaced entangled state of two sideband modes of an optical beam is used for the initial quantum resource to create entanglement between two atomic ensembles. To extend this method to multipartite entanglement more sidebands with different frequency shifts have to be prepared and the number of entangled sideband modes must be strictly restricted by the bandwidth of optical parametric amplifier, which is the device for generating optical entangled state in their system. On the other hand, it is difficult to spatially separate these entangled optical submodes with small frequency intervals. Although a narrow band optical cavity can be utilized for separating these optical modes, entanglement among them will be significantly reduced^36^. These limitations make difficult to extend the experimental method of ref. ^35^ to entangle more atomic ensembles. Up to now, it still remains a challenge to entangle more than two remote quantum memories in CV regime.

Here we present an experimental demonstration on deterministically establishing, storing, and releasing of CV entanglement among three atomic ensembles. At first, a tripartite optical entangled state is off-line prepared, and then the entanglement is transferred into three atomic ensembles located 2.6 m apart from each other via electromagnetically induced transparency (EIT) interaction. After a given storage time, the preserved atomic entanglement is controllably released into three separated quantum channels consisting of three entangled optical submodes. The dependence of entanglement among three released optical submodes on systematic parameters is theoretically deduced and multipartite entanglement transfer as well as storage are experimentally proved. Since the tripartite optical entangled state is generated by linearly optical transformation of three squeezed states of light, its three submodes are naturally space separated^37, 38^. The presented scheme can be directly extended to generate optical entangled states with more submodes if more squeezed states of light are available. In this way, entanglement of more atomic ensembles can be established.

## Results

### Experimental set-up

**Fig. 1:**
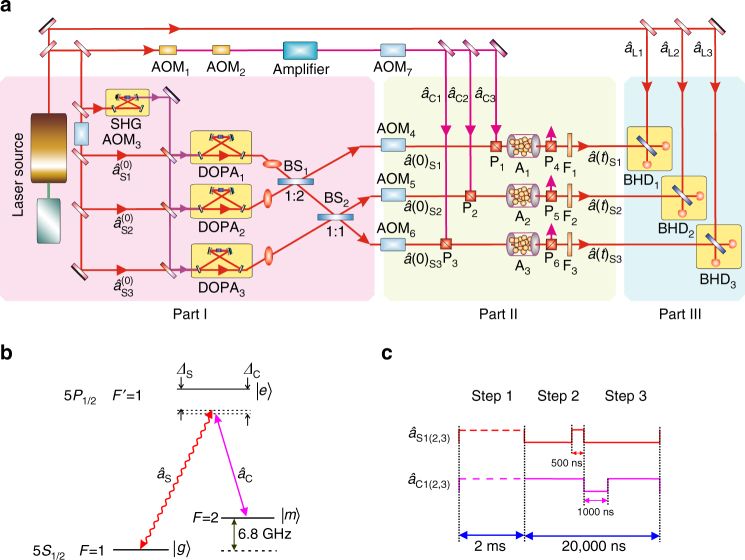
Schematic diagram. **a** Experimental set-up. It includes three parts, Part I is the generation system of tripartite optical entanglement; Part II expresses the transportation of entanglement of optical modes to three distant atomic ensembles; Part III is the entanglement verification system. A_1–3_, atomic ensemble_1–3_; DOPA_1–3_, degenerate optical parametric amplifier_1–3_; SHG, second harmonic generator; AOM_1–7_, acousto-optical modulator_1–7_; BS_1–2_, beam splitter_1–2_; P_1–6_, Glan–Thompson polarizer_1–6_; F_1–3_, filter_1–3_; BHD_1–3_, balanced homodyne detector_1–3_; Amplifier, laser amplifier. **b** ^87^Rb atomic-level configuration and relevant transitions. $\left| {5{S_{1/2}},F = 1} \right\rangle$ and $\left| {5{S_{1/2}},F = 2} \right\rangle$ play the roles of ground state $\left| g \right\rangle$ and meta-state $\left| m \right\rangle$, respectively, and $\left| {5{P_{1/2}},F\prime = 1} \right\rangle$ is the excited state $\left| e \right\rangle$. Classical control optical beam ${\hat a_{\rm{C}}}$ (*solid line*) and quantum probe optical beam ${\hat a_{\rm{S}}}$ (*wavy line*) are shown. **c** Experimental time sequence for control optical beams ${\hat a_{{\rm{C1(2,3)}}}}$ and signal optical beams ${\hat a_{{\rm{S1(2,3)}}}}$

Figure 1a describes the experimental set-up for generation, storage, and transfer of tripartite entanglement. Three space separated submodes $\hat a{(0)_{{\rm{S1}}}}$, $\hat a{(0)_{{\rm{S2}}}},$ and $\hat a{(0)_{{\rm{S3}}}}$ of an optical entangled state off-line prepared in Part I interact, respectively, with three atomic memories A_1_, A_2_, and A_3_ located at three distant nodes to generate and store entanglement of spin waves among three atomic ensembles. Then the preserved entanglement is transferred back into an optical entangled state with three submodes $\hat a{(t)_{{\rm{S1}}}}$, $\hat a{(t)_{{\rm{S2}}}},$ and $\hat a{(t)_{{\rm{S3}}}}$ after a storage time *t* (Part II). At last, entanglement among three released optical submodes is measured by three balanced homodyne detectors BHD_1–3_ (Part III).

Three narrow band entangled optical beams tuned to the $\left| {5{S_{1/2}},F = 1} \right\rangle$ ↔ $\left| {5{P_{1/2}},F' = 1} \right\rangle$ transition of rubidium around 795 nm are obtained via linearly optical transformation of three squeezed states of light, which are generated by three degenerate optical parametric amplifiers (DOPA_1–3_). DOPA_1_ and DOPA_2(3)_ operating in parametric amplification and deamplification produce phase and amplitude quadrature squeezed states ${\hat a_{{\rm{S1}}}}$ and ${\hat a_{{\rm{S2(3)}}}}$, respectively^39, 40^. The squeezing parameters (*r*) for the three squeezed states are assumed to be identical for simplicity. In fact, three DOPAs used in our experiment have totally identical configuration and thus their squeezing parameters are almost the same. The quadrature amplitudes ${\hat X_{{\rm{S}}j}} = ( {{{\hat a}_{{\rm{S}}j}} + \hat a_{{\rm{S}}j}^ + } ){\rm{/}}\sqrt 2$ and phases ${\hat P_{{\rm{S}}j}} = ( {{{\hat a}_{{\rm{S}}j}} - \hat a_{{\rm{S}}j}^ + } ){\rm{/}}\sqrt 2 i$ (*j* = 1, 2, 3) of output optical beams from three DOPAs are expressed as ${\hat X_{{\rm{S}}1}} = {{\rm{e}}^r}\hat X_{{\rm{S}}1}^{(0)}$, ${\hat P_{{\rm{S}}1}} = {{\rm{e}}^{ - r}}\hat P_{{\rm{S}}1}^{(0)}$, ${\hat X_{{\rm{S}}2(3)}} = {{\rm{e}}^{ - r}}\hat P_{{\rm{S}}1}^{(0)},$ and ${\hat P_{{\rm{S}}2(3)}} = {{\rm{e}}^r}\hat P_{{\rm{S}}2(3)}^{(0)}$ ^40^, where $\hat X(\hat P)_{{\rm{S}}1}^{(0)}$, $\hat X(\hat P)_{{\rm{S}}2}^{(0)},$ and $\hat X(\hat P)_{{\rm{S}}3}^{(0)}$ are amplitude (phase) quadratures of input optical beams $\hat a_{{\rm{S}}1}^{(0)}$, $\hat a_{{\rm{S}}2}^{(0)},$ and $\hat a_{{\rm{S}}3}^{(0)}$ for DOPAs, respectively. By interfering three squeezed states of light on BS_1_ and BS_2_, we obtain a tripartite optical entangled state with quantum correlations of both amplitude quadratures $\hat X{(0)_{{\rm{L}}j}} =( {\hat a{{(0)}_{{\rm{S}}j}} + \hat a(0)_{{\rm{S}}j}^ + }){\rm{/}}\sqrt 2$ and phase quadratures $\hat P{(0)_{{\rm{L}}j}} =( {\hat a{{(0)}_{{\rm{S}}j}} - \hat a(0)_{{\rm{S}}j}^ + }){\rm{/}}\sqrt 2 i$ ^40^. Then the three entangled optical beams are chopped into 500 ns pulses $\hat a{(0)_{{\rm{S}}1}}$, $\hat a{(0)_{{\rm{S}}2}},$ and $\hat a{(0)_{{\rm{S}}3}}$, with three acoustical–optical modulators AOM_4–6_, which are used for the input signals of three atomic ensembles, i.e., ^87^Rb vapor cells A_1_, A_2_, and A_3_.

### Quantum state transfer

The physical mechanism of light–matter interaction used for the experiment is EIT, which is a transparency phenomenon induced by optical field in an opaque medium by means of quantum interference^41^. M. Fleischhauer and M.D. Lukin have theoretically demonstrated that when quantum fields propagate in EIT media, there are form-stable quantum excitations associated with such propagation, named dark-state polaritons, and in this process the quantum state of light can be ideally transferred to collective atomic excitations and vice versa. Therefore, EIT effects can be applied to generate nonclassical states of atomic ensembles, to store optical quantum states, and reversibly to release stored quantum states into optical channels, respectively^42–45^. An atomic ensemble is represented by total angular momentum operator of collective atomic spins $\hat J = \mathop {\sum}\nolimits_i \left| g \right\rangle \left\langle m \right|$, and *y*, *z*-components of the collective atomic angular momentum play the role of canonical variables, i.e., ${\hat X_{\rm{A}}} = ( {\hat J + {{\hat J}^ + }} ){\rm{/}}\sqrt 2 = {\hat J_y}{\rm{/}} \sqrt {\langle {{\hat J}_x} \rangle }$, ${\hat P_{\rm{A}}} = \left( {\hat J - {{\hat J}^ + }} \right){\rm{/}}\sqrt 2 i = {\hat J_z}{\rm{/}}\sqrt {\left\langle {{{\hat J}_x}} \right\rangle }$ ^35^. When control optical beams are adiabatically switched off, the mapping relations of amplitude (phase) quadratures from input optical submodes $\hat X\left( {\hat P} \right){(0)_{{\rm{L}}j}}$ to atomic spin waves $\hat X\left( {\hat P} \right){(t)_{{\rm{A}}j}}$ after a storage time *t* are expressed by refs.^46, 47^:

1 $$\begin{array}{l} \hat X{(t)_{{\rm{A}}j}} = \sqrt {{\eta _{_{\rm{M}}}}} \hat X{(0)_{{\rm{L}}j}} + \sqrt {1 - {\eta _{_{\rm{M}}}}} \hat X_{{\rm{A}}j}^{{\rm{vac}}}{\rm{,}}\\ \hat P{(t)_{{\rm{A}}j}} = \sqrt {{\eta _{_{\rm{M}}}}} \hat P{(0)_{{\rm{L}}j}} + \sqrt {1 - {\eta _{_{\rm{M}}}}} \hat P_{{\rm{A}}j}^{{\rm{vac}}}{\rm{,}} \end{array}$$

where the mapping efficiency from input optical submodes to atomic spin waves is ${\eta _{_{\rm{M}}}} = {\eta _{_{\rm{T}}}}{\eta _{_{\rm{W}}}}{{\rm{e}}^{ - t/{\tau _{\rm{s}}}}}$, *η*_T_ is the optical transmission efficiency, *η*_W_ is the storage efficiency of light in atomic ensemble, and *τ*_s_ is the storage lifetime limited by atomic decoherence. The vacuum quadrature noises of atomic ensembles $\hat X\left( {\hat P} \right)_{{\rm{A}}j}^{{\rm{vac}}}$ are introduced by limited mapping efficiency *η*_M_. Since canonical quadrature operators of atomic spin waves obey the same commutation relation with that of Gaussian optical states, i.e., $\left[ {{{\hat X}_{\rm{A}}},{{\hat P}_{\rm{A}}}} \right] = i$, using similar procedure of deducing full tripartite inseparability criteria provided by Loock et al.^48^, we can obtain a set of analogous criterion inequalities for atomic spin waves (see also the “Methods” section).

The stored atomic entanglement can be transferred to tripartite entanglement among three output optical submodes $\hat a{(t)_{{\rm{S}}1}}$, $\hat a{(t)_{{\rm{S}}2}},$ and $\hat a{(t)_{{\rm{S}}3}}$ after a storage time *t* by turning on control optical beams. The quadrature amplitudes and phases of released submodes, $\hat X{(t)_{{\rm{L}}j}} =( {\hat a{{(t)}_{{\rm{S}}j}} + \hat a(t)_{{\rm{S}}j}^ + }){\rm{/}}\sqrt 2$ and $\hat P{(t)_{{\rm{L}}j}} =( {\hat a{{(t)}_{{\rm{S}}j}} - \hat a(t)_{{\rm{S}}j}^ + }){\rm{/}}\sqrt 2 i$ in terms of quadratures for atomic spin waves $\hat X\left( {\hat P} \right){(t)_{{\rm{A}}j}}$ are expressed by refs. ^46, 47^:

2 $$\begin{array}{l} \hat X{(t)_{{\rm{L}}j}} = - \sqrt {\eta {'_{\rm{M}}}} \hat X{(t)_{{\rm{A}}j}} + \sqrt {1 - \eta {'_{\rm{M}}}} \hat X_{{\rm{L}}j}^{{\rm{vac}}}{\rm{,}}\\ \hat P{(t)_{{\rm{L}}j}} = - \sqrt {\eta {'_{\rm{M}}}} \hat P{(t)_{{\rm{A}}j}} + \sqrt {1 - \eta {'_{\rm{M}}}} \hat P_{{\rm{L}}j}^{{\rm{vac}}}{\rm{,}}\\ \end{array}$$

where the mapping efficiency from atomic spin waves to optical submodes $\eta {'_{_{\rm{M}}}}$ is the retrieval efficiency from atomic ensembles to light. The vacuum quadrature noises of optical submodes $\hat X\left( {\hat P} \right)_{{\rm{L}}j}^{{\rm{vac}}}$ are introduced by the read process.

The full tripartite inseparability criteria for released optical modes are given by ref.^48^:

3 $$\begin{array}{*{20}{l}} {I{{(t)}_{{\rm{L}}1}}} \hfill & = \hfill & {\left\langle {{\delta ^2}\left( {\hat X{{(t)}_{{\rm{L}}2}} - \hat X{{(t)}_{{\rm{L}}3}}} \right)} \right\rangle {\rm{/}}2 + \left\langle {{\delta ^2}\left( {g{'_{{\rm{L}}1}}\hat P{{(t)}_{{\rm{L}}1}}} \right.} \right.} \hfill \\ {} \hfill & {} \hfill & {\left. {\left. { + \hat P{{(t)}_{{\rm{L}}2}} + \hat P{{(t)}_{{\rm{L}}3}}} \right)} \right\rangle {\rm{/}}2\geq 1{\rm{,}}} \hfill \\ {I{{(t)}_{{\rm{L}}2}}} \hfill & = \hfill & {\left\langle {{\delta ^2}\left( {\hat X{{(t)}_{{\rm{L}}1}} - \hat X{{(t)}_{{\rm{L}}3}}} \right)} \right\rangle {\rm{/}}2 + \left\langle {{\delta ^2}\left( {\hat P{{(t)}_{{\rm{L}}1}}} \right.} \right.} \hfill \\ {} \hfill & {} \hfill & {\left. {\left. { + g{'_{{\rm{L}}2}}\hat P{{(t)}_{{\rm{L}}2}} + \hat P{{(t)}_{{\rm{L}}3}}} \right)} \right\rangle {\rm{/}}2\geq 1{\rm{,}}} \hfill \\ {I{{(t)}_{{\rm{L}}3}}} \hfill & = \hfill & {\left\langle {{\delta ^2}\left( {\hat X{{(t)}_{{\rm{L}}1}} - \hat X{{(t)}_{{\rm{L}}2}}} \right)} \right\rangle {\rm{/}}2 + \left\langle {{\delta ^2}\left( {\hat P{{(t)}_{{\rm{L}}1}}} \right.} \right.} \hfill \\ {} \hfill & {} \hfill & {\left. {\left. { + \hat P{{(t)}_{{\rm{L}}2}} + g{'_{{\rm{L}}3}}\hat P{{(t)}_{{\rm{L}}3}}} \right)} \right\rangle {\rm{/}}2\geq 1.} \hfill \end{array}$$

**Fig. 2:**
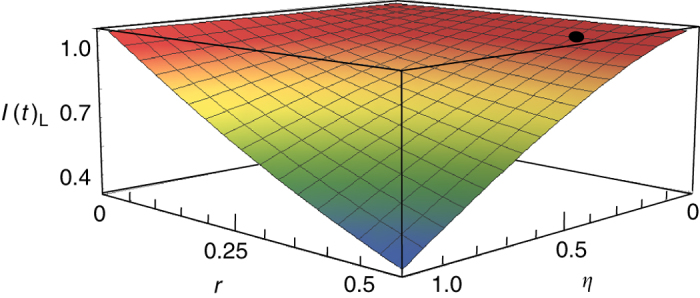
The dependence of combinations of normalized quantum correlation variances among three released submodes after a storage time of 1000 ns on the squeezing parameter *r* of three DOPAs and the total mapping efficiency *η*, where the gains $g{\prime _{{\rm{L1}} - {\rm{L3}}}}$ are taken as the optimal gain $g\prime_{{\rm{L1}} - {\rm{L3}}}^{{\rm{opt}}}$. The *dot* corresponds the experimental result of *I*(*t*)_L_ = 0.96 ± 0.01, where the squeezing parameter *r* is 0.38 and total mapping efficiency *η* is about 16%

If any two in the three inequalities are simultaneously violated, the three submodes form a tripartite Greenberger–Horne–Zeilinger-like (GHZ-like) entangled state of light, where $g{'_{{\rm{L1}}}}$, $g{'_{{\rm{L2}}}},$ and $g{'_{{\rm{L3}}}}$ are the gain factors for optimizing the correlation variances for released submodes. From Eqs. (1) and (2), it can be seen that entanglement is limited by total mapping efficiencies *η* $( {\eta = {\eta _{_{\rm{M}}}}\eta {'_{_{\rm{M}}}}} )$ as well as squeezing parameter *r*. When the squeezing parameters *r* for three DOPAs and the total mapping efficiencies *η* for three atomic memories are the same, the values of left sides of three inequalities (3) are identical, *I*(*t*)_L1_ = *I*(*t*)_L2_ = *I*(*t*)_L3_ = *I*(*t*)_L_. The smaller *I*(*t*)_L_ is, the higher the entanglement is. Figure 2 shows the dependence of correlation variance combinations for three released submodes on the squeezing parameter *r* of initial squeezed states and the total mapping efficiency *η* with the storage time of 1000 ns. We can see that the combinations of correlation variance are reduced with the increase of squeezing parameter *r* and total mapping efficiency *η* (see also the “Methods” section).

When the control optical beams ${\hat a_{{\rm{C1}}}}$, ${\hat a_{{\rm{C2}}}},$ and ${\hat a_{{\rm{C3}}}}$, which are tuned to $\left| {5{S_{1/2}},F = 2} \right\rangle$ ↔ $\left| {5{P_{1/2}},F' = 1} \right\rangle$ transition of rubidium, are adiabatically switched off, optical entanglement among three input submodes $\hat a{(0)_{{\rm{S1}}}}$, $\hat a{(0)_{{\rm{S2}}}},$ and $\hat a{(0)_{{\rm{S3}}}}$ is transferred to atomic entanglement of spin waves according to Eq. (1) via EIT interaction. After a storage time of 1000 ns, control optical beams ${\hat a_{{\rm{C1}}}}$, ${\hat a_{{\rm{C2}}}},$ and ${\hat a_{{\rm{C3}}}}$ are switched on again, three optical submodes $\hat a{(t)_{{\rm{S1}}}}$, $\hat a{(t)_{{\rm{S2}}}},$ and $\hat a{(t)_{{\rm{S3}}}}$ are released. The combinations of correlation variances in inequalities (3) are measured with three time domain BHD_1–3_. The intensive coherent light ${\hat a_{{\rm{L1}}}}$, ${\hat a_{{\rm{L2}}}},$ and ${\hat a_{{\rm{L3}}}}$ are utilized as local oscillators of BHD_1–3_. The control and signal optical beams with orthogonally linear polarizations are combined on Glan–Thompson polarizers (P_1–3_) before atomic cells, and control optical beams are filtered out from the signal optical beams by Glan–Thompson polarizers (P_4–6_) and etalon filters (F_1–3_). In storage and retrieval procedures 10,000 traces of BHD output signals with 20 G samples/s are digitally filtered with a bandpass filter of 2.5 MHz and averaged to obtain the optimal entangled degree. In this case, the low-frequency sideband noises resulting from pumping laser of DOPAs and atomic ensembles, as well as high frequency noises coming from parametric conversion in DOPAs and EIT process in atomic ensembles have been filtered out. When both control and signal optical beams are blocked and only the local oscillators are remained, outputs of BHDs stand for the corresponding vacuum noise level^49^.

### Experimental results

**Fig. 3:**
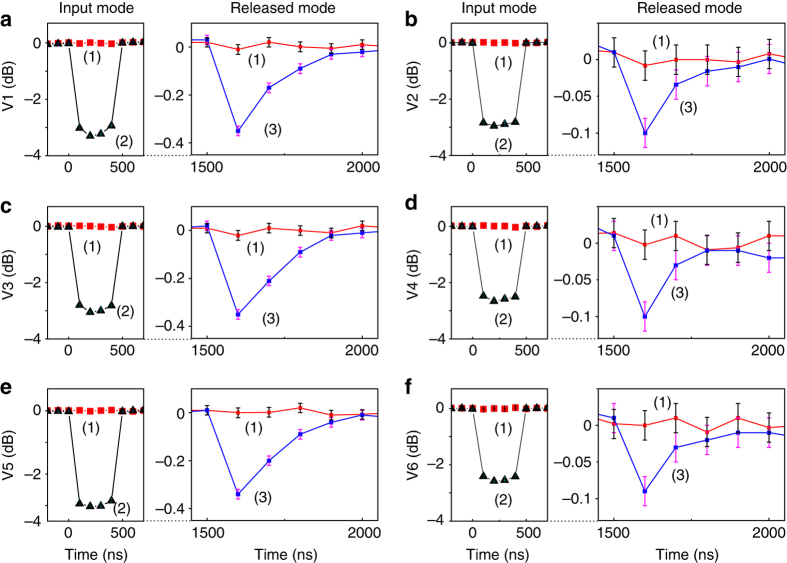
Measured normalized correlation variances of input and released optical submodes. Trace (1) is the vacuum noise level. Trace (2) is the correlation variances of the original input optical submodes. **a** V1 $( {\langle {{\delta ^2}( {\hat X{{(0)}_{{\rm{L2}}}} - \hat X{{(0)}_{{\rm{L3}}}}})} \rangle })$, **b** V2 $( {\langle {{\delta ^2}( {g_{{\rm{L1}}}^{{\rm{opt}}}\hat P{{(0)}_{{\rm{L1}}}} + \hat P{{(0)}_{{\rm{L2}}}} + \hat P{{(0)}_{{\rm{L3}}}}})}\rangle })$, **c** V3 $( {\langle {{\delta ^2}( {\hat X{{(0)}_{{\rm{L1}}}} - \hat X{{(0)}_{{\rm{L3}}}}})}\rangle })$, **d** V4 $( {\langle {{\delta ^2}( {\hat P{{(0)}_{{\rm{L1}}}} + g_{{\rm{L2}}}^{{\rm{opt}}}\hat P{{(0)}_{{\rm{L2}}}} + \hat P{{(0)}_{{\rm{L3}}}}})}\rangle })$, **e** V5 $( {\langle {{\delta ^2}( {\hat X{{(0)}_{{\rm{L1}}}} - \hat X{{(0)}_{{\rm{L2}}}}})}\rangle })$, **f** V6 $( {\langle {{\delta ^2}( {\hat P{{(0)}_{{\rm{L1}}}} + \hat P{{(0)}_{{\rm{L2}}}} + g_{{\rm{L3}}}^{{\rm{opt}}}\hat P{{(0)}_{{\rm{L3}}}}})}\rangle })$. Trace (3) is corresponding correlation variances of released optical submodes after a storage time of 1000 ns. *Error bars* represent ±1 standard error and are obtained with the statistics of the measured correlation variances

The normalized correlation variances for different combinations of quadrature components are given in Table. 1, where gain factors (*g*_1_, *g*_2,_ and *g*_3_) are chosen as the optimal gains for minimizing the corresponding correlation variances. The correlation variances of input and released submodes are directly measured with three sets of BHDs. The normalized correlation variances among three atomic ensembles are inferred from Eq. (2), where the mapping efficiency $\eta {'_{_{\rm{M}}}}$ is about 68% for our experimental system. The measured normalized quantum correlation variances are shown in Fig. 3. The squeezing parameter *r* is 0.38 and total mapping efficiency *η* is about 16%. The combination of correlation variances for three released submodes is *I*(*t*)_L_ = 0.96 ± 0.01, which is less than 1, thus according to criterion inequalities (3) the entanglement among released submodes is verified. The value is in agreement with the theoretically calculated result, which is marked with a *black dot* in Fig. 2. Because of the limitation of total mapping efficiency, the correlation variances of released entangled state are much higher than that of input state. However, correlation variances below the corresponding vacuum noise level in Fig. 3 and the violation of criterion inequalities (3) certainly prove the existence of tripartite entanglement among three optical submodes released from atomic spin waves of three atomic ensembles. Thus the tripartite GHZ-like entanglement among three atomic ensembles is experimentally demonstrated.

**Table 1:** The values of normalized correlation variances for different combinations

Correlation variances for different combinations	Values for input submodes (dB)	Values for atomic spin waves (dB)	Values for released submodes (dB)
$\langle {{\delta ^2}( {{{\hat X}_2} - {{\hat X}_3}})}\rangle$	−3.30 ± 0.05	−0.56 ± 0.03	−0.37 ± 0.03
$\langle {{\delta ^2}( {{g_1}{{\hat P}_1} + {{\hat P}_2} + {{\hat P}_3}})}\rangle$	−2.93 ± 0.05	−0.15 ± 0.02	−0.10 ± 0.02
$\langle {{\delta ^2}( {{{\hat X}_1} - {{\hat X}_3}})}\rangle$	−3.25 ± 0.05	−0.53 ± 0.03	−0.35 ± 0.03
$\langle {{\delta ^2}( {{{\hat P}_1} + {g_2}{{\hat P}_2} + {{\hat P}_3}})}\rangle$	−2.91 ± 0.05	−0.15 ± 0.02	−0.10 ± 0.02
$\langle {{\delta ^2}( {{{\hat X}_1} - {{\hat X}_2}} )} \rangle$	−3.25 ± 0.05	−0.52 ± 0.03	−0.34 ± 0.03
$\langle {{\delta ^2}( {{{\hat P}_1} + {{\hat P}_2} + {g_3}{{\hat P}_3}} )} \rangle$	−2.90 ± 0.05	−0.14 ± 0.02	−0.09 ± 0.02

## Discussion

In summary, deterministic quantum entanglement among three spatially separated quantum nodes is experimentally generated, stored, and transferred. Within storage lifetime, the multipartite entanglement is preserved in three space separated atomic ensembles, and then at a desirable time the stored atomic entanglement can be controllably converted into three optical submodes to be quantum channels. Our work shows that multipartite CV entanglement can be established among remote macroscopic objects by transferring off-line prepared entanglement of optical beams into atomic ensembles via EIT interaction. Since unconditional CV entanglement among multipartite optical modes has been experimentally accomplished^37, 50, 51^, mature quantum optical technology can be used for realizing entanglement of more nodes in a quantum networks. The obtained entanglement among atomic ensembles depends on mapping efficiency and initial squeezing parameter. The higher the squeezing and the mapping efficiency are, the better the entanglement among atomic ensembles is. In the presented experimental system, the total mapping efficiency is mainly limited by the optical transmission loss and memory (storage and retrieval) efficiency. The transmission loss (about 18%) comes from the optical losses of atomic cells, etalon filters, Glan–Thompson polarizers and other optical components, which can be further reduced if better optical elements with lower losses are available.

The increased excess noises in released submodes from memories originate from fluorescence and coherent emission as well as spurious fluctuations in signal channels induced by the control optical beam^44^. On the other hand, since both EIT and four-wave mixing (FWM) effects are simultaneously generated in an ensemble of hot atoms^52^, the control optical beam acts as a far-detuned field on the signal transition in the undesired FWM process and spontaneously generates an “ idler” field, which also can form excess noises. These mechanisms resulting in excess noises always exist in EIT light–matter interaction for any input state (vacuum state, squeezed vacuum state, entangled state or others). Therefore, some schemes used for improving EIT memory efficiency of classical signals^52–54^, such as decreasing detuning of probe and control optical beams, increasing the power of control optical beam and enhancing the temperature of atomic vapor, will unavoidably introduce more excess noises into atomic media and reduce quantum correlations among atomic ensembles. However, by optimizing experimental parameters, excess noises can be minimized for a given EIT experimental system^43, 44, 52^.

It has been demonstrated that mapping efficiency can be significantly improved by means of the technology of optical cavity enhancement without introducing excess noise^55, 56^ and that the storage lifetime can be dramatically increased if thermal atomic ensembles are replaced by cold atoms confined in three-dimensional optical lattice^55^. Generation systems of optical squeezed states with squeezing up to 15 dB are available today^57^, providing initial high quality quantum resources for establishing better multipartite entanglement among atomic memories. The presented scheme opens up a new possibility for constructing future quantum internet^1^ and implementing distributed quantum computation based on the use of deterministic CV entanglement resources of light and atomic memories^58^.

## Methods

### Tripartite entangled state of light

A Ti:sapphire laser (Coherent MBR-110) pumped by green laser (Yuguang DPSS FG-VIIIB) outputs 3 W laser at about 795 nm, which is used for pumping light of second harmonic generator (SHG) and seed lights of DOPAs. The configuration of optical cavities for SHG and three DOPAs is identical bow tie-type ring cavity with a 1 × 2 × 10 mm periodically poled KTiOPO4 (PPKTP) crystal. Three DOPAs are pumped by the second harmonic fields at about 398 nm from SHG and the fundamental waves from SHG are utilized as three injected seed fields $\hat a_{{\rm{S1}}}^{(0)}$, $\hat a_{{\rm{S2}}}^{(0)},$ and $\hat a_{{\rm{S3}}}^{(0)}$. DOPA_1_ and DOPA_2(3)_ are operated at parametric amplification and deamplification to produce quadrature phase- and amplitude-squeezed state of light, respectively^8^. The three squeezed optical beams are interfered on two optical beam splitters. The quadrature phase-squeezed field from DOPA_1_ $\left( {{{\hat a}_{{\rm{S1}}}}} \right)$ and the quadrature amplitude-squeezed field from DOPA_2_ $\left( {{{\hat a}_{{\rm{S2}}}}} \right)$ are first interfered on a beam splitter (BS_1_) with the ratio of R:T = 1:2 (R: reflectivity and T: transmissivity). Then, one of two output optical beams from BS_1_ and the quadrature amplitude-squeezed light from DOPA_3_ $\left( {{{\hat a}_{{\rm{S3}}}}} \right)$ are interfered on BS_2_ with the ratio of R:T = 1:1. The relative phase between the two input optical beams on BS_1(2)_ is kept at 2*kπ* (*k* is integer). Finally, three entangled optical beams are chopped into three optical pulses $\hat a{(0)_{{\rm{S1}}}}$, $\hat a{(0)_{{\rm{S2}}}},$ and $\hat a{(0)_{{\rm{S3}}}}$ by three AOM_4–6_. The three optical pulses are, respectively, injected into three atomic ensembles to be the input optical submodes. The quadrature’s amplitudes and phases of input optical submodes are expressed by ref. ^40^:

4 $$\begin{array}{l} \hat X{(0)_{{\rm{L}}1}} = \sqrt {\frac{1}{3}} {{\rm{e}}^r}\hat X_{{\rm{S}}1}^{(0)} + \sqrt {\frac{2}{3}} {{\rm{e}}^{ - r}}\hat X_{{\rm{S}}2}^{(0)}{\rm{,}}\\ \hat P{(0)_{{\rm{L}}1}} = \sqrt {\frac{1}{3}} {{\rm{e}}^{ - r}}\hat P_{{\rm{S}}1}^{(0)} + \sqrt {\frac{2}{3}} {{\rm{e}}^r}\hat P_{{\rm{S}}2}^{(0)}{\rm{,}}\\ \hat X{(0)_{{\rm{L}}2}} = \sqrt {\frac{1}{3}} {{\rm{e}}^r}\hat X_{{\rm{S}}1}^{(0)} - \sqrt {\frac{1}{6}} {{\rm{e}}^{ - r}}\hat X_{{\rm{S}}2}^{(0)} + \sqrt {\frac{1}{2}} {{\rm{e}}^{ - r}}\hat X_{{\rm{S}}3}^{(0)}{\rm{,}}\\ \hat P{(0)_{{\rm{L}}2}} = \sqrt {\frac{1}{3}} {{\rm{e}}^{ - r}}\hat P_{{\rm{S}}1}^{(0)} - \sqrt {\frac{1}{6}} {{\rm{e}}^r}\hat P_{{\rm{S}}2}^{(0)} + \sqrt {\frac{1}{2}} {{\rm{e}}^r}\hat P_{{\rm{S}}3}^{(0)}{\rm{,}}\\ \hat X{(0)_{{\rm{L}}3}} = \sqrt {\frac{1}{3}} {{\rm{e}}^r}\hat X_{{\rm{S}}1}^{(0)} - \sqrt {\frac{1}{6}} {{\rm{e}}^{ - r}}\hat X_{{\rm{S}}2}^{(0)} - \sqrt {\frac{1}{2}} {{\rm{e}}^{ - r}}\hat X_{{\rm{S}}3}^{(0)}{\rm{,}}\\ \hat P{(0)_{{\rm{L}}3}} = \sqrt {\frac{1}{3}} {{\rm{e}}^{ - r}}\hat P_{{\rm{S}}1}^{(0)} - \sqrt {\frac{1}{6}} {{\rm{e}}^r}\hat P_{{\rm{S}}2}^{(0)} - \sqrt {\frac{1}{2}} {{\rm{e}}^r}\hat P_{{\rm{S}}3}^{(0)}{\rm{,}}\end{array}$$

respectively.

The inequalities of full inseparability criteria for input tripartite entangled states of light are ref. ^48^:

5 $$\begin{array}{l} I{(0)_{{\rm{L}}1}} = \left\langle {{\delta ^2}\left( {\hat X{{(0)}_{{\rm{L}}2}} - \hat X{{(0)}_{{\rm{L}}3}}} \right)} \right\rangle {\rm{/}}2 + \left\langle {{\delta ^2}\left( {{g_{{\rm{L}}1}}\hat P{{(0)}_{{\rm{L}}1}} + \hat P{{(0)}_{{\rm{L}}2}} + \hat P{{(0)}_{{\rm{L}}3}}} \right)} \right\rangle {\rm{/}}2\geq 1{\rm{,}}\\ I{(0)_{{\rm{L}}2}} = \left\langle {{\delta ^2}\left( {\hat X{{(0)}_{{\rm{L}}1}} - \hat X{{(0)}_{{\rm{L}}3}}} \right)} \right\rangle {\rm{/}}2 + \left\langle {{\delta ^2}\left( {\hat P{{(0)}_{{\rm{L}}1}} + {g_{{\rm{L}}2}}\hat P{{(0)}_{{\rm{L}}2}} + \hat P{{(0)}_{{\rm{L}}3}}} \right)} \right\rangle {\rm{/}}2\geq 1{\rm{,}}\\ I{(0)_{{\rm{L}}3}} = \left\langle {{\delta ^2}\left( {\hat X{{(0)}_{{\rm{L}}1}} - \hat X{{(0)}_{{\rm{L}}2}}} \right)} \right\rangle {\rm{/}}2 + \left\langle {{\delta ^2}\left( {\hat P{{(0)}_{{\rm{L}}1}} + \hat P{{(0)}_{{\rm{L}}2}} + {g_{{\rm{L}}3}}\hat P{{(0)}_{{\rm{L}}3}}} \right)} \right\rangle {\rm{/}}2\geq 1.\end{array}$$

If any two in the three inequalities are simultaneously violated, the three submodes form a tripartite GHZ-like entangled state, where *g*_L1_, *g*_L2_, and *g*_L3_ are gain factors for minimizing correlation variances of the input tripartite entangled state of light.

When the squeezing parameter *r* $\left( {r\geq 0} \right)$ for three DOPAs is the same, the gain factors in inequalities (5) should be the same, i.e., *g*_L1_ = *g*_L2_ = *g*_L3_ = *g*_L_, and the values of left sides of three inequalities are identical, i.e., *I*(0)_L1_ = *I*(0)_L2_ = *I*(0)_L3_ = *I*(0)_L_. Using Eq. (4), the combination of normalized quantum correlation variances for input optical beams is obtained:

6 $$I{(0)_{\rm{L}}} = \frac{{12{{\rm{e}}^{ - 2r}} + 2{{\left( {{g_{\rm{L}}} + 2} \right)}^2}{{\rm{e}}^{ - 2r}} + 4{{\left( {{g_{\rm{L}}} - 1} \right)}^2}{{\rm{e}}^{2r}}}}{{24}}{\rm{.}}$$

Calculating the minimum value of Eq. (6), we get the optimal gain factor $g_{\rm{L}}^{{\rm{opt}}}$:

7 $$g_{\rm{L}}^{{\rm{opt}}} = \frac{{2{{\rm{e}}^{4r}} - 2}}{{2{{\rm{e}}^{4r}} + 1}}{\rm{.}}$$

If squeezing parameter *r* is larger than 0, the combination of correlation variances will be less than 1 with the optimal gain factor, and the input optical submodes of atomic ensembles are in a tripartite GHZ-like entangled state.

### Establishing tripartite atomic ensemble entanglement

In EIT memory medium, quantum state can be mapped from input optical submode $\hat a{(0)_{\rm{S}}}$ into atomic spin wave $\hat J$ and vice versa under the interaction with a strong control optical beam ${\hat a_{\rm{C}}}$ ^41, 42^. The control optical beam is treated as classical optical beam *A*_C_ because it is much more intensive than the signal optical modes. In EIT process, the effective interaction Hamiltonian ${\hat H_{{\rm{EIT}}}}$ between signal optical mode $\hat a{(0)_{\rm{S}}}$ and atomic spin wave $\hat J$ is given by refs. ^2, 46^:

8 $${\hat H_{{\rm{EIT}}}} = i\hbar \kappa {A_{\rm{C}}}\hat a{(0)_{\rm{S}}}{\hat J^ + } - i\hbar \kappa {A_{\rm{C}}}\hat a(0)_{\rm{S}}^ + \hat J{\rm{,}}$$

which is similar to a beam splitter interaction, where *κ* stands for the interaction constant between light and atoms.

By solving Heisenberg motion equations with the Hamiltonian ${\hat H_{{\rm{EIT}}}}$ (Eq. 8), we obtain the expressions of quantum storage process and the mapping relations (Eq. 1) of amplitude and phase quadratures from input optical submodes $\hat X\left( {\hat P} \right){(0)_{{\rm{L}}j}}$ to atomic spin waves $\hat X\left( {\hat P} \right){(t)_{{\rm{A}}j}}$ after a storage time *t*.

When the control optical beams are turned on, the input signal submodes are compressed in atomic ensembles due to the slow propagation under EIT interaction. On the moment of simultaneously shutting off three control optical beams, quantum entanglement among three pulse submodes will be mapped into atomic spin waves in the three ensembles, where the control optical beam plays the role of writing process. Using Eqs. (1) and (4), the amplitude (phase) quadratures of atomic spin waves $\hat X\left( {\hat P} \right){(t)_{{\rm{A}}j}}$ after a storage time *t* are obtained:

9 $$\begin{array}{l} \hat X{(t)_{{\rm{A}}1}} = \sqrt {\frac{{{\eta _{_{\rm{M}}}}}}{3}} {{\rm{e}}^r}\hat X_{{\rm{S}}1}^{(0)} + \sqrt {\frac{{2{\eta _{_{\rm{M}}}}}}{3}} {{\rm{e}}^{ - r}}\hat X_{{\rm{S}}2}^{(0)} + \sqrt {1 - {\eta _{_{\rm{M}}}}} \hat X_{{\rm{A}}1}^{{\rm{vac}}}{\rm{,}}\\ \hat P{(t)_{{\rm{A}}1}} = \sqrt {\frac{{{\eta _{_{\rm{M}}}}}}{3}} {{\rm{e}}^{ - r}}\hat P_{{\rm{S}}1}^{(0)} + \sqrt {\frac{{2{\eta _{_{\rm{M}}}}}}{3}} {{\rm{e}}^r}\hat P_{{\rm{S}}2}^{(0)} + \sqrt {1 - {\eta _{_{\rm{M}}}}} \hat P_{{\rm{A}}1}^{{\rm{vac}}}{\rm{,}}\\ \hat X{(t)_{{\rm{A}}2}} = \sqrt {\frac{{{\eta _{_{\rm{M}}}}}}{3}} {{\rm{e}}^r}\hat X_{{\rm{S}}1}^{(0)} - \sqrt {\frac{{{\eta _{_{\rm{M}}}}}}{6}} {{\rm{e}}^{ - r}}\hat X_{{\rm{S}}2}^{(0)} + \sqrt {\frac{{{\eta _{_{\rm{M}}}}}}{2}} {{\rm{e}}^{ - r}}\hat X_{{\rm{S}}3}^{(0)} + \sqrt {1 - {\eta _{_{\rm{M}}}}} \hat X_{{\rm{A}}2}^{{\rm{vac}}}{\rm{,}}\\ \hat P{(t)_{{\rm{A}}2}} = \sqrt {\frac{{{\eta _{_{\rm{M}}}}}}{3}} {{\rm{e}}^{ - r}}\hat P_{{\rm{S}}1}^{(0)} - \sqrt {\frac{{{\eta _{_{\rm{M}}}}}}{6}} {{\rm{e}}^r}\hat P_{{\rm{S}}2}^{(0)} + \sqrt {\frac{{{\eta _{_{\rm{M}}}}}}{2}} {{\rm{e}}^r}\hat P_{{\rm{S}}3}^{(0)} + \sqrt {1 - {\eta _{_{\rm{M}}}}} \hat P_{{\rm{A}}2}^{{\rm{vac}}}{\rm{,}}\\ \hat X{(t)_{{\rm{A}}3}} = \sqrt {\frac{{{\eta _{_{\rm{M}}}}}}{3}} {{\rm{e}}^r}\hat X_{{\rm{S}}1}^{(0)} - \sqrt {\frac{{{\eta _{_{\rm{M}}}}}}{6}} {{\rm{e}}^{ - r}}\hat X_{{\rm{S}}2}^{(0)} - \sqrt {\frac{{{\eta _{_{\rm{M}}}}}}{2}} {{\rm{e}}^{ - r}}\hat X_{{\rm{S}}3}^{(0)} + \sqrt {1 - {\eta _{_{\rm{M}}}}} \hat X_{{\rm{A}}3}^{{\rm{vac}}}{\rm{,}}\\ \hat P{(t)_{{\rm{A}}3}} = \sqrt {\frac{{{\eta _{_{\rm{M}}}}}}{3}} {{\rm{e}}^{ - r}}\hat P_{{\rm{S}}1}^{(0)} - \sqrt {\frac{{{\eta _{_{\rm{M}}}}}}{6}} {{\rm{e}}^r}\hat P_{{\rm{S}}2}^{(0)} - \sqrt {\frac{{{\eta _{_{\rm{M}}}}}}{2}} {{\rm{e}}^r}\hat P_{{\rm{S}}3}^{(0)} + \sqrt {1 - {\eta _{_{\rm{M}}}}} \hat P_{{\rm{A}}3}^{{\rm{vac}}}{\rm{,}} \end{array}$$

respectively.

Since canonical quadrature operators of atomic spin waves obey the same commutation relation with that of Gaussian optical states, i.e., $\left[ {{{\hat X}_{\rm{A}}},{{\hat P}_{\rm{A}}}} \right] = i$, using similar procedure of deducing full tripartite inseparability criteria provided by Loock et al.^48^, we obtain a set of analogous criterion inequalities for atomic spin waves:

10 $$\begin{array}{l} I{(t)_{{\rm{A1}}}} = \left\langle {{\delta ^2}\left( {\hat X{{(t)}_{{\rm{A2}}}} - \hat X{{(t)}_{{\rm{A3}}}}} \right)} \right\rangle {\rm{/}}2 + \left\langle {{\delta ^2}\left( {{g_{{\rm{A1}}}}\hat P{{(t)}_{{\rm{A1}}}} + \hat P{{(t)}_{{\rm{A2}}}} + \hat P{{(t)}_{{\rm{A3}}}}} \right)} \right\rangle {\rm{/}}2\geq 1{\rm{,}}\\ I{(t)_{{\rm{A2}}}} = \left\langle {{\delta ^2}\left( {\hat X{{(t)}_{{\rm{A1}}}} - \hat X{{(t)}_{{\rm{A3}}}}} \right)} \right\rangle {\rm{/}}2 + \left\langle {{\delta ^2}\left( {\hat P{{(t)}_{{\rm{A1}}}} + {g_{{\rm{A2}}}}\hat P{{(t)}_{{\rm{A2}}}} + \hat P{{(t)}_{{\rm{A3}}}}} \right)} \right\rangle {\rm{/}}2\geq 1{\rm{,}}\\ I{(t)_{{\rm{A3}}}} = \left\langle {{\delta ^2}\left( {\hat X{{(t)}_{{\rm{A1}}}} - \hat X{{(t)}_{{\rm{A2}}}}} \right)} \right\rangle {\rm{/}}2 + \left\langle {{\delta ^2}\left( {\hat P{{(t)}_{{\rm{A1}}}} + \hat P{{(t)}_{{\rm{A2}}}} + {g_{{\rm{A3}}}}\hat P{{(t)}_{{\rm{A3}}}}} \right)} \right\rangle {\rm{/}}2\geq 1. \end{array}$$

When any two in three inequalities are violated, the three atomic ensembles are in an entangled state of GHZ-like type, where *g*_A1_, *g*_A2_, and *g*_A3_ are gain factors for atomic ensembles.

If the mapping efficiencies *η*_M_ for three atomic memories are the same, the gain factors in inequalities (10) should be the same, i.e., *g*_A1_ = *g*_A2_ = *g*_A3_ = *g*_A_, and the values of three inequalities are also identical, i.e., *I*(*t*)_A1_ = *I*(*t*)_A2_ = *I*(*t*)_A3_ = *I*(*t*)_A_. According to Eq. (9), the combination of normalized quantum correlation variances for atomic spin waves after a storage time *t* is obtained:

11 $$I{(t)_{\rm{A}}} = \frac{{12{{\rm{e}}^{ - 2r}} + 2{{\left( {{g_{\rm{A}}} + 2} \right)}^2}{{\rm{e}}^{ - 2r}} + 4{{\left( {{g_{\rm{A}}} - 1} \right)}^2}{{\rm{e}}^{2r}}}}{{24}}{\eta _{_{\rm{M}}}} + \left( {1 + \frac{1}{4}{g_{\rm{A}}}} \right)\left( {1 - {\eta _{_{\rm{M}}}}} \right){\rm{.}}$$

Similarly, by minimizing *I*(*t*)_A_ we get the optimal gain factor $g_{\rm{A}}^{{\rm{opt}}}$:

12 $$g_{\rm{A}}^{{\rm{opt}}} = \frac{{2{\eta _{_{\rm{M}}}}{{\rm{e}}^{4r}} - 2{\eta _{_{\rm{M}}}}}}{{3{{\rm{e}}^{2r}} + {\eta _{_{\rm{M}}}} - 3{\eta _{_{\rm{M}}}}{{\rm{e}}^{2r}} + 2{{\rm{e}}^{4r}}{\eta _{_{\rm{M}}}}}}{\rm{.}}$$

**Fig. 4:**
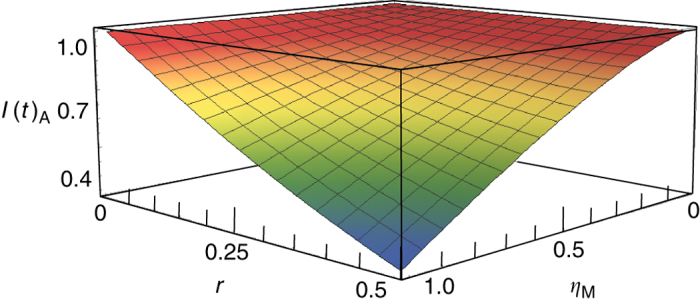
The dependence of combination of normalized quantum correlation variances among three atomic ensembles after a storage time of 1000 ns on the squeezing parameter *r* of three DOPAs and the mapping efficiency *η*_M_, where the gains are taken as the optimal gain $g_{{\rm{A1}} - {\rm{A3}}}^{{\rm{opt}}}$

The smaller *I*(*t*)_A_ is, the better the atomic entanglement is. Figure 4 shows the dependence of correlation variance combination for three atomic ensembles on the squeezing parameter *r* of initial squeezed states and the mapping efficiency *η*_M_ with the storage time of 1000 ns. We can see that the combination is reduced with the increase of squeezing parameter *r* and mapping efficiency *η*_M_. In our experiment, the squeezing parameter *r* and the mapping efficiency *η*_M_ are about 0.38 and 23%, respectively.

### Quantum state transfer of stored entangled state

The control optical beams of read process enable to map quantum state from the atomic spin waves to released optical submodes. Using Eqs. (2) and (9), the amplitude (phase) quadratures $\hat X\left( {\hat P} \right){(t)_{{\rm{L}}j}}$ of released optical submodes after a storage time *t* are calculated. We have:

13 $$\begin{array}{l} \hat X{(t)_{{\rm{L}}1}} = \sqrt {\frac{\eta }{3}} {{\rm{e}}^r}\hat X_{{\rm{S}}1}^{(0)} + \sqrt {\frac{{2\eta }}{3}} {{\rm{e}}^{ - r}}\hat X_{{\rm{S}}2}^{(0)} + \sqrt {1 - \eta } \hat X_{{\rm{L}}1}^{{\rm{vac}}}{\rm{,}}\\ \hat P{(t)_{{\rm{L}}1}} = \sqrt {\frac{\eta }{3}} {{\rm{e}}^{ - r}}\hat P_{{\rm{S}}1}^{(0)} + \sqrt {\frac{{2\eta }}{3}} {{\rm{e}}^r}\hat P_{{\rm{S}}2}^{(0)} + \sqrt {1 - \eta } \hat P_{{\rm{L}}1}^{{\rm{vac}}}{\rm{,}}\\ \hat X{(t)_{{\rm{L}}2}} = \sqrt {\frac{\eta }{3}} {{\rm{e}}^r}\hat X_{{\rm{S}}1}^{(0)} - \sqrt {\frac{\eta }{6}} {{\rm{e}}^{ - r}}\hat X_{{\rm{S}}2}^{(0)} + \sqrt {\frac{\eta }{2}} {{\rm{e}}^{ - r}}\hat X_{{\rm{S}}3}^{(0)} + \sqrt {1 - \eta } \hat X_{{\rm{L}}2}^{{\rm{vac}}}{\rm{,}}\\ \hat P{(t)_{{\rm{L}}2}} = \sqrt {\frac{\eta }{3}} {{\rm{e}}^{ - r}}\hat P_{{\rm{S}}1}^{(0)} - \sqrt {\frac{\eta }{6}} {{\rm{e}}^r}\hat P_{{\rm{S}}2}^{(0)} + \sqrt {\frac{\eta }{2}} {{\rm{e}}^r}\hat P_{{\rm{S}}3}^{(0)} + \sqrt {1 - \eta } \hat P_{{\rm{L}}2}^{{\rm{vac}}}{\rm{,}}\\ \hat X{(t)_{{\rm{L}}3}} = \sqrt {\frac{\eta }{3}} {{\rm{e}}^r}\hat X_{{\rm{S}}1}^{(0)} - \sqrt {\frac{\eta }{6}} {{\rm{e}}^{ - r}}\hat X_{{\rm{S}}2}^{(0)} - \sqrt {\frac{\eta }{2}} {{\rm{e}}^{ - r}}\hat X_{{\rm{S}}3}^{(0)} + \sqrt {1 - \eta } \hat X_{{\rm{L}}3}^{{\rm{vac}}}{\rm{,}}\\ \hat P{(t)_{{\rm{L}}3}} = \sqrt {\frac{\eta }{3}} {{\rm{e}}^{ - r}}\hat P_{{\rm{S}}1}^{(0)} - \sqrt {\frac{\eta }{6}} {{\rm{e}}^r}\hat P_{{\rm{S}}2}^{(0)} - \sqrt {\frac{\eta }{2}} {{\rm{e}}^r}\hat P_{{\rm{S}}3}^{(0)} + \sqrt {1 - \eta } \hat P_{{\rm{L}}3}^{{\rm{vac}}}{\rm{.}} \end{array}$$

In this case of $g{'_{{\rm{L}}1}} = g{'_{{\rm{L}}2}} = g{'_{{\rm{L}}3}} = g{'_{\rm{L}}}$, *I*(*t*)_L_ in inequalities (3) can be calculated from Eq. (13):

14 $$I{(t)_{\rm{L}}} = \frac{{12{{\rm{e}}^{ - 2r}} + 2{{\left( {g{'_{\rm{L}}} + 2} \right)}^2}{{\rm{e}}^{ - 2r}} + 4{{\left( {g{'_{\rm{L}}} - 1} \right)}^2}{{\rm{e}}^{2r}}}}{{24}}\eta + \left( {1 + \frac{1}{4}g{'_{\rm{L}}}} \right)(1 - \eta ).$$

The optimal gain factor $\left( {g\prime_{\rm{L}}^{{\rm{opt}}}} \right)$ for read out process equals to:

15 $$g\prime_{\rm{L}}^{{\rm{opt}}} = \frac{{2\eta {{\rm{e}}^{4r}} - 2\eta {'_{_{\rm{M}}}}}}{{3{{\rm{e}}^{2r}} + \eta - 3\eta {{\rm{e}}^{2r}} + 2{{\rm{e}}^{4r}}\eta }}{\rm{.}}$$

Figure 2 is obtained from Eq. (14), where the optimal gain in Eq. (15) is applied.

### Atomic ensemble

The atomic energy levels of rubidium used for quantum memory medium is illustrated in Fig. 3b. The collective coherence of the ground state $\left| {5{S_{1/2}},F = 1} \right\rangle$ and meta-state $\left| {5{S_{1/2}},F = 2} \right\rangle$ is used to store nonclassical state of light. For balancing the storage efficiency and excess noises from atoms, the frequencies of both signal and control optical beams are detuned by *Δ*_s_ = 700 MHz (the detuning of signal optical beam from the transition between energy levels g and e) and *Δ*_c_ = 700.5 MHz (the detuning of control optical beam from the transition between energy levels m and e), respectively. The detuning is realized by two sets of double-pass 1.7 GHz acousto-optical modulators (AOM_1–2_). Three rubidium vapor cells of 7.5 cm-long with 10 torr of neon buffer gas in a *μ* metal magnetic shielding are used as atomic media, where the neon buffer gas prevents thermal diffusion to increase the atomic coherence. The three rubidium atomic cells are heated to around 65 °C in our experiment.

### Experimental time sequence

In the beginning of each period, the laser is turned on for 2 ms by AOM_3_ and split into three optical beams $\hat a_{{\rm{S1}}}^{(0)}$, $\hat a_{{\rm{S2}}}^{(0)},$ and $\hat a_{{\rm{S3}}}^{(0)}$, which are used as input signals of DOPAs, respectively, for persistently locking the length of optical cavities. Then the input signals of DOPAs are turned off for 20,000 ns by AOM_3_ to generate three squeezed vacuum states of light. Within the locking period the three entangled optical submodes are chopped into 500 ns pulses with AOM_4–6_, which are used for input submodes $\hat a{(0)_{{\rm{S1}}}}$, $\hat a{(0)_{{\rm{S2}}}},$ and $\hat a{(0)_{{\rm{S3}}}}$ of three atomic ensembles. Once the signal pulses enter into the atomic cell, the control optical beams ${\hat a_{{\rm{C1}}}}$, ${\hat a_{{\rm{C2}}}},$ and ${\hat a_{{\rm{C3}}}}$ are switched off by AOM_7_ to complete the quantum storage. At a user-controlled moment (1000 ns for our experiment) within storage lifetime, the control optical beams ${\hat a_{{\rm{C1}}}}$, ${\hat a_{{\rm{C2}}}},$ and ${\hat a_{{\rm{C3}}}}$ are turned on again by AOM_7_ at to obtain three released optical pulses $\hat a{(t)_{{\rm{S1}}}}$, $\hat a{(t)_{{\rm{S2}}}},$ and $\hat a{(t)_{{\rm{S3}}}}$.

### Data availability

The data that support the findings of this study are available from the corresponding author on request.
